# Supplementary material for: Barriers and facilitators to self-care practices for sexual and reproductive health among women of reproductive age
Source: PLoS One. 2024 May 22;19(5):e0303958. doi: 10.1371/journal.pone.0303958 (PMC11111034; doi:10.1371/journal.pone.0303958)
Supplement: S1 File — (PDF) [file pone.0303958.s001.pdf]

Informed consent form

Barriers and facilitators to self-care practices for sexual and reproductive health among women of reproductive age

<u>Description:</u>

This research *Barriers and facilitators to self-care practices for sexual and reproductive health among women of reproductive age* aims to explore the barriers and facilitators in adopting self-care practices for sexual and reproductive health (SRH) among women of reproductive age. Through qualitative exploration, the study seeks to understand knowledge and utilization of various self-care methods among married or child-bearing women. Your insights in this study are pivotal for current policy and program discussion and explore effective strategies to enhance access to evidence-based self-care programs and methods, thereby empowering women and girls to take control of their sexual and reproductive health and rights.

We would like to assure of anonymity and confidentiality of your personal information and identity throughout the research process. While no remuneration is provided for participation, individuals have the autonomy to decline answering any questions that they find challenging or uncomfortable. The duration of the interview is expected to range from 35 to 45 minutes, during which participants will have the opportunity to share their perspectives and experiences regarding self-care practices for SRH. The information and data provided by you will be used for the research report and manuscript publication purpose ensuring confidentiality. We would like to request your support and consent to participate in this study.

[For participants]

I fully understand the title, objective, scope and provide my wholehearted consent for the interview and to publish the data.

Name of participant:

Signature:

Date:

Barriers and facilitators to self-care practices for sexual and reproductive health among women of reproductive age

Guiding interview question for women practicing SRH self-care

**Interview number:**
**Name of the participants**
**Age:**
**Gender/Sexuality:**
**Religion:**
**Marital status:**
**Date of interview:**
**Consent status:**
**Interview time:**

Self-management:

1. Any self-medication that you take or self-treatment that you perform? If there is any need for you that you can remember?
2. Are you aware of how to perform a self-examination" , what is it used for ? If there is any need for you that you can remember?
3. Could you please take us through any facilitators and barriers of the self-management process based on your experience?

Self testing

4. Have you used pregnancy test kit or HIV/ STI testing kit or FP devices? What do you think is benefit or harm of such kit and devices?
5. Could you please share your experience of monitoring the menstrual cycle or pregnancy period

6. What facilitates and hinders self-testing process for you?

Self-awareness:

7. Could you please share your experience of helping yourself in regards to sexual and reproductive health?
8. Do you have prior information or did you look for information relating to HIV and AIDS and STIs/ maternal health/ sexual health/ hormones/FP devices? Where do you look?
9. Do you have self-regulation practice for use of contraceptives, testing kits, intake of hormones, visit to clinic for regular check-up, keep track of your appointment? Do you use any digital device? Have you taken service from toll free number or hotline numbers from any institutions?
10. What can be done to enhance the quality of self-care intervention?

Guiding interview question for managers/advocates/service providers

Interview number:

Name of the participants

Age:

Gender/Sexuality:

Religion:

Marital status:

Date of interview:

Consent status:

Self-management:

1. Any self-medication or self-treatment or self-examination services that your institution is currently providing or advocating for your beneficiary? If not, do you think there is any need of self-medication or self-treatment or self-examination services that your beneficiary want or you deem necessary? (Probe for self-injecting drugs)

Self-testing:

2. What do you think about the benefits or harms of using self-testing facilities such as pregnancy test kit or HIV testing kit?
3. Are there any self-sampling, self-screening, self-diagnosis, self-collection, self-monitoring mechanism for your client? If not, do you think it is necessary for your client?

Self-awareness:

4. In your opinion what could be the importance of self-help, self-awareness, and self-regulation?
5. Do you have prior information, or did you look for information relating to HIV and AIDS and STIs/ maternal health/ sexual health/ hormones/FP devices?
6. Could you please share the available programs to promote self-awareness among your beneficiaries?

Policy implications:

7. What are current policy level and administration challenges in development of self-care intervention targeting self-management, self-testing and self-awareness?
8. What are current policy level and administration challenges in delivery of self-care intervention targeting self-management, self-testing and self-awareness?
9. In your experience and understanding what enhances access to quality, evidence-based self-care interventions for SRHR in Nepal?
10. Have your organization promoted self-care initiative or advocated for it? Can you share your experiences regarding this?
