# Supplementary material for: Barriers and facilitators to self-care practices for sexual and reproductive health among women of reproductive age
Source: PLoS One. 2024 May 22;19(5):e0303958. doi: 10.1371/journal.pone.0303958 (PMC11111034; doi:10.1371/journal.pone.0303958)
Supplement: Attachment — Submitted filename: Response letter_PONE-D-23-31576 (1).docx [file pone.0303958.s002.docx]

**28-03-2024**

**Oluwatosin Oluwaseun Olu-Abiodun**

Academic Editor, PLOS ONE

**RE: Revision of Manuscript B-4942-Manuscript-33258**

Dear Editor,

We would like to thank you and the reviewers for your time and for providing constructive comments on our manuscript entitled "**Barriers and facilitators to self-care practices for sexual and reproductive health among women of reproductive age."**

A revised version (clean and track changes) reflecting the point-by-point response to the reviewers’ comments has been submitted for your consideration. We believe we have addressed all the comments and the manuscript has significantly improved and ready for publication in your esteemed journal.

Should you require any additional information, please do not hesitate to contact me. Thank you very much for your time and kind consideration.

We look forward to getting our manuscript published soon.

Your sincerely,

**Amit Timilsina**

**RESPONSE TO THE REVIEWERS**

Journal Requirements:

When submitting your revision, we need you to address these additional requirements.

1. **Please ensure that your manuscript meets PLOS ONE's style requirements, including those for file naming. The PLOS ONE style templates can be found at**
   <https://journals.plos.org/plosone/s/file?id=wjVg/PLOSOne_formatting_sample_main_body.pdf> and 
   <https://journals.plos.org/plosone/s/file?id=ba62/PLOSOne_formatting_sample_title_authors_affiliations.pdf>

**RESPONSE:** The style requirement of Plos One has been reviewed and all necessary adjustments have been made.

**2. Note from Emily Chenette, Editor in Chief of PLOS ONE, and Iain Hrynaszkiewicz, Director of Open Research Solutions at PLOS: Did you know that depositing data in a repository is associated with up to a 25% citation advantage (**[**https://doi.org/10.1371/journal.pone.0230416**](https://doi.org/10.1371/journal.pone.0230416)**)? If you’ve not already done so, consider depositing your raw data in a repository to ensure your work is read, appreciated and cited by the largest possible audience. You’ll also earn an Accessible Data icon on your published paper if you deposit your data in any participating repository (**[**https://plos.org/open-science/open-data/#accessible-data**](https://plos.org/open-science/open-data/#accessible-data)**).**

**RESPONSE:** The raw data are context and issue sensitive thus to respect the confidentiality and anonymity of respondent, the raw data cannot be shared open. However, if the data is required for anyone, it can be made available upon direct request to the first author at timilsinaamit@gmail.com.

**3. We note that your Data Availability Statement is currently as follows: All relevant data are within the manuscript and its supporting informations.**

Please confirm at this time whether or not your submission contains all raw data required to replicate the results of your study. Authors must share the “minimal data set” for their submission. PLOS defines the minimal data set to consist of the data required to replicate all study findings reported in the article, as well as related metadata and methods (<https://journals.plos.org/plosone/s/data-availability#loc-minimal-data-set-definition>).

For example, authors should submit the following data:

- The values behind the means, standard deviations and other measures reported;
- The values used to build graphs;
- The points extracted from images for analysis.

Authors do not need to submit their entire data set if only a portion of the data was used in the reported study.

If your submission does not contain these data, please either upload them as Supporting Information files or deposit them to a stable, public repository and provide us with the relevant URLs, DOIs, or accession numbers. For a list of recommended repositories, please see <https://journals.plos.org/plosone/s/recommended-repositories>.

If there are ethical or legal restrictions on sharing a de-identified data set, please explain them in detail (e.g., data contain potentially sensitive information, data are owned by a third-party organization, etc.) and who has imposed them (e.g., an ethics committee). Please also provide contact information for a data access committee, ethics committee, or other institutional body to which data requests may be sent. If data are owned by a third party, please indicate how others may request data access.

**RESPONSE:** The codebook produced as the outcome of the data analysis, the interview guideline for the IDI and KII, and the informed consent forms have been available for this study. As participants have not consented to the availability of full interview transcripts during this study, it has not publicly available to respect participants' anonymity and consent. However, if any additional data is required, it can be made available upon direct request to the data repository at [info@asiapacificalliance.org](mailto:info@asiapacificalliance.org) respecting the confidentiality and anonymity of the participants.

**4. Please include your full ethics statement in the ‘Methods’ section of your manuscript file. In your statement, please include the full name of the IRB or ethics committee who approved or waived your study, as well as whether or not you obtained informed written or verbal consent. If consent was waived for your study, please include this information in your statement as well.**

**RESPONSE:** This section has been updated in Methodology ( Line: 134-136)

Changes: The Nepal Health Research Council (Ref. No: 3093) reviewed the research proposal provided ethical clearance for the study, and approved the study. The research follows standard ethical principles guided by the Helsinki Ethics Principles. The autonomy and confidentiality of participants have been maintained throughout the project period. All the participants were oriented about the study and its importance. Before data collection, informed written consent was taken from the participants after orientation, verbal consent was taken before starting the interviews and their right to withdraw from the research was reiterated. The participants were assured of anonymity and confidentiality before, during and after the study.

**5. Please review your reference list to ensure that it is complete and correct. If you have cited papers that have been retracted, please include the rationale for doing so in the manuscript text, or remove these references and replace them with relevant current references. Any changes to the reference list should be mentioned in the rebuttal letter that accompanies your revised manuscript. If you need to cite a retracted article, indicate the article’s retracted status in the References list and also include a citation and full reference for the retraction notice.**

**RESPONSE: We have double-checked the reference list, edited and ensured that it is complete and correct.**

**Reviewers' comments:**

5. Review Comments to the Author

Please use the space provided to explain your answers to the questions above. You may also include additional comments for the author, including concerns about dual publication, research ethics, or publication ethics. (Please upload your review as an attachment if it exceeds 20,000 characters)

**Reviewer #1: The manuscript was presented with a technically sound piece of scientific research backed with data. The data provided supports the conclusions. The conclusions drawn appropriately based on the data presented. The language used for submission was clear.**

**RESPONSE: We would like to thank the reviewer for appreciating our work.**

**Reviewer #2: The manuscript is technically sound, and the data supports the conclusion. However, the conclusion could be improved to reflect/capture all sub-themes that emerged in the study according to their main theme (SRH self-care practices, facilitators, and barriers).**
**RESPONSE: Thank you for your suggestion. The revised conclusion in the abstract and the main text include the study's key findings/themes**.

Discussion: all sub-themes could be discussed
Line 221: Limited access should be separated from poor knowledge as a sub-heading for discussion.
**RESPONSE:** We have revised the discussion section and tried to include the most relevant sub-themes. The sub-heading “limited access” has been separated according to reviewer’s suggestion.

Line 107: self-care practices or experiences. Which variable are the authors studying? The word should be operationalized for clarity.
Data from service providers was not reported. (Line 328 reports)

**RESPONSE:** Our interest is in self-care practices and operationalized in the methods. For consistency, we have used the phrase, self-care practices, throughout the main text.

Page no. Lines 290, 357, and 379 include data from the service providers (health care providers), while lines 344 and 405 provide data for the community workers. The service providers mentioned in this study include both healthcare providers and community health workers.
